# Supplementary material for: Spindle motility skews division site determination during asymmetric cell division in Physcomitrella
Source: Nat Commun. 2022 May 5;13:2488. doi: 10.1038/s41467-022-30239-1 (PMC9072379; doi:10.1038/s41467-022-30239-1)
Supplement: Supplementary file 1 — Supplementary Information [file 41467_2022_30239_MOESM1_ESM.pdf]

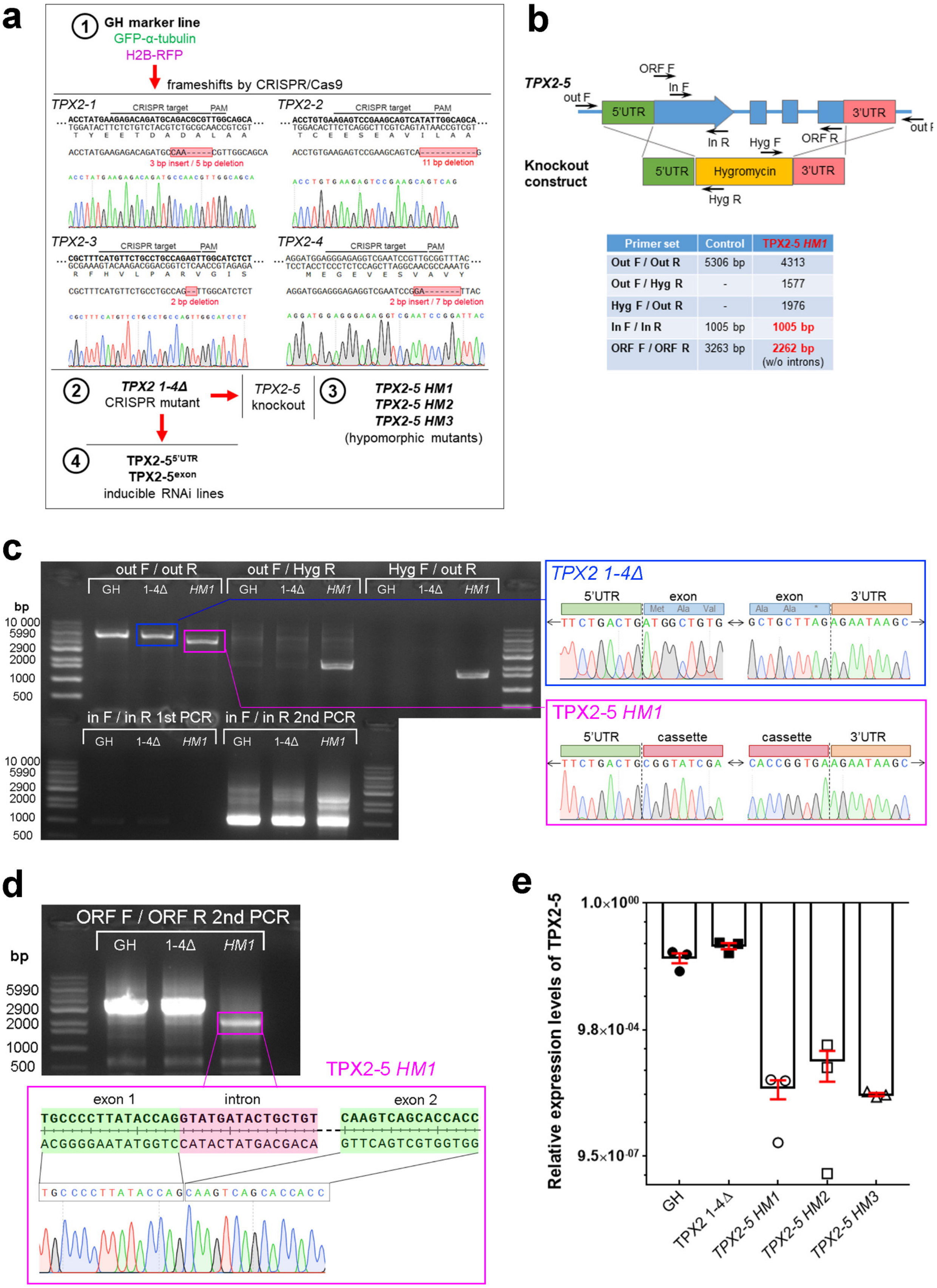

Supplementary Figure 1. Isolation of hypomorphic *TPX2-5* mutants

(a) Schematic explanation of *P. patens* lines created and used in this study and representative sequencing data of frameshift mutations in the *TPX2 1-4Δ* line. **(b)** Schematic explanation of *TPX2-5* hypomorphic line selection and genotyping PCR. **(c)** Results of genotyping PCR and sequencing of the *TPX2-5* locus. **(d)** Results of amplifying full coding sequence from start to stop codons and sequencing results of the *TPX2-5* gene. Note the band shift in the *TPX2-5 HM1* line due to loss of introns. Experiment was repeated 3 times with similar results. **(e)** qRT-PCR analysis of *TPX2-5* expression normalized against the expression of the internal housekeeping genes *EF1α* and *L21*. Bars represent mean values ± SEM from *n* = 3 independent experiments.

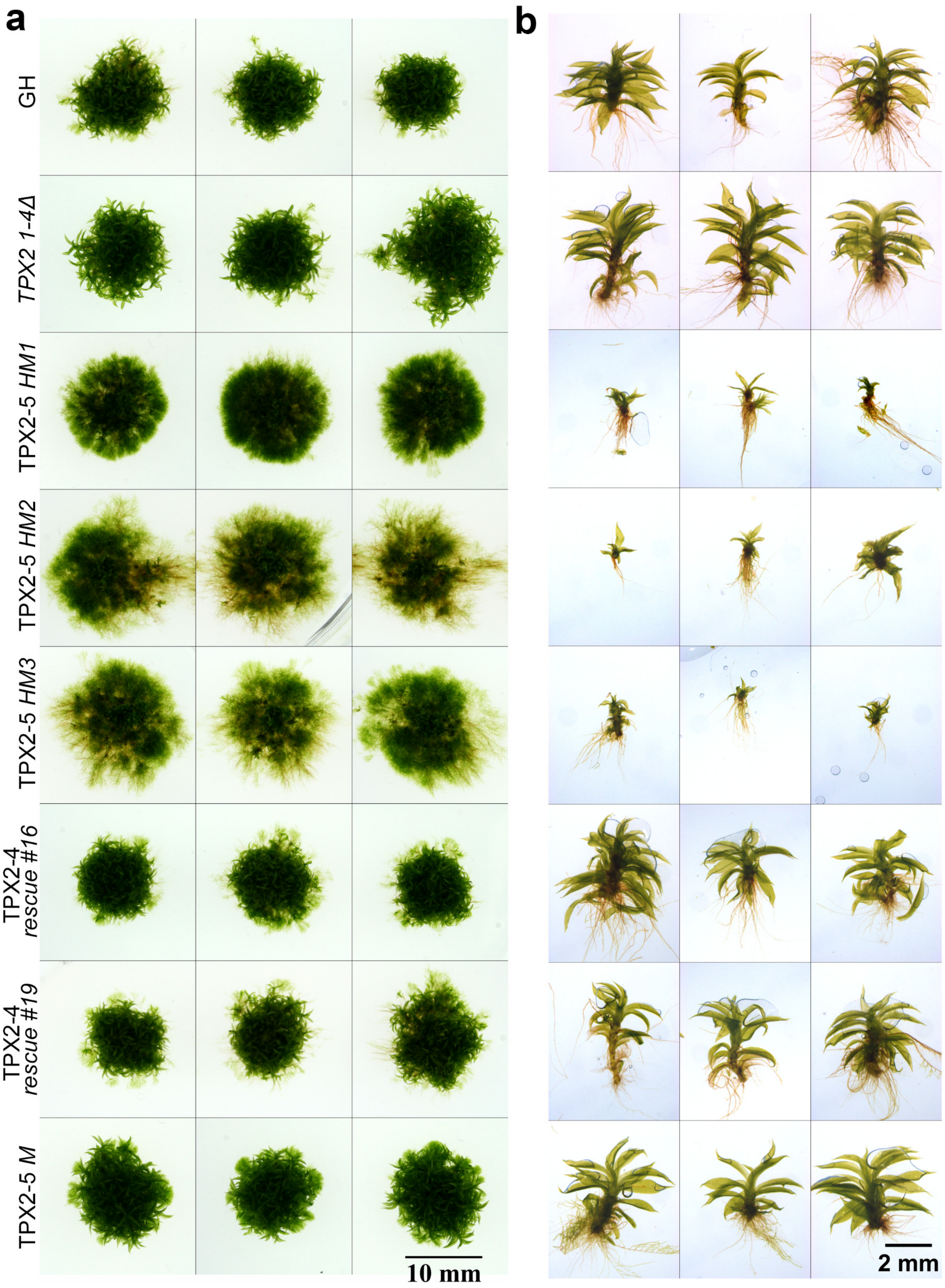

Supplementary Figure 2. Development of *P. patens* colonies and gametophores after 4 weeks of culture

Representative images of moss colonies **(a)** or gametophores **(b)** for all lines (except RNAi) used in this study after 4 weeks of culture on BCDAT agar plates. The brightness and contrast were linearly adjusted. Experiment was repeated 3 times with similar results. Bars, 10 mm and 2 mm, respectively.

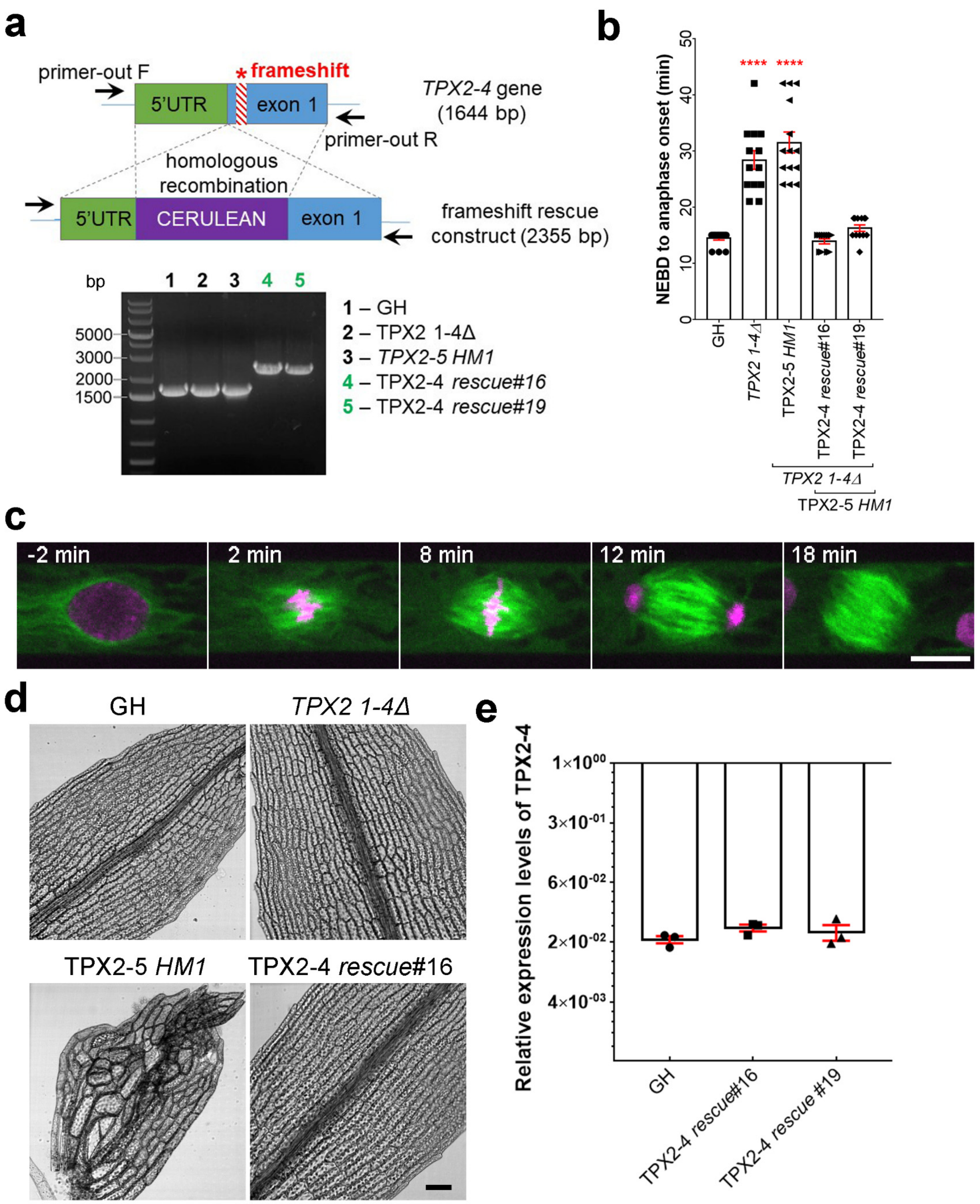

Supplementary Figure 3. Rescue of the *TPX2-5 HM1* phenotypes by frameshift rescue of the *TPX2-4* gene

(a) Schematic illustration of the frameshift rescue experiment. In brief, the N-terminus-coding region of the *TPX2-4* gene in the *TPX2-5 HM1* background was tagged with Cerulean flanked with ~500 bp of the 5′-UTR and exon region (without the frameshift mutation) by homologous recombination. Construct integration was verified by PCR. Experiment was repeated 2 times with similar results. **(b)** Mitotic duration of protonemal cells calculated from NEBD to anaphase onset in GH (control, $n = 16$ cells), *TPX2 1-4Δ* ($n = 13$), *TPX2-5 HM1* ($n = 14$), and two independent *TPX2-4* rescue lines #16 and #19 ($n = 11$ and 12, respectively). Bars represent mean ± SEM, ****p=0.0001 by one-way Anova with Dunnett's multiple comparison test against GH. **(c)** Mitotic progression in the *TPX2-4* rescue #16 line. Note that perinuclear MTs and prometaphase spindle formation were restored. Time 0 (min) was set at NEBD. Live-cell imaging was repeated 2 times with similar results. Bar, 10 µm. **(d)** Representative images of gametophore leaf cells in GH, *TPX2 1-4Δ*, *TPX2-5 HM*, and *TPX2-4 rescue*#16 lines. Imaging was performed 2 times with similar results. Bar, 100 µm. **(e)** qRT-PCR analysis of *TPX2-4* expression normalized against the expression of the internal housekeeping genes *EF1α* and *L21*. No significant difference between GH and *TPX2-4 rescue*#16 and #19 by one-way Anova test with Dunnett's multiple comparison test and two-tailed Student's t-test. Bars represent mean ± SEM from $n = 3$ independent experiments.

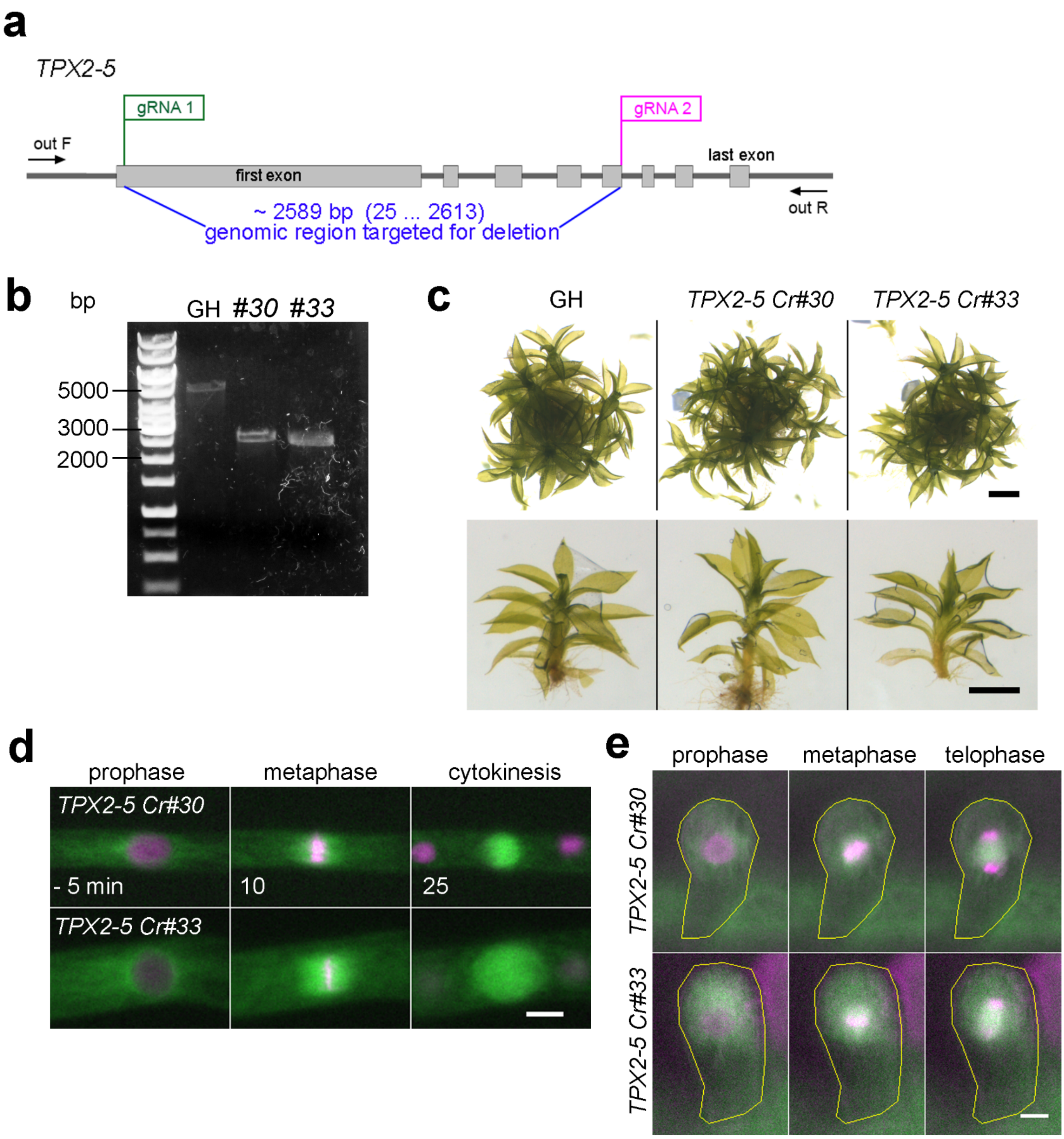

Supplementary Figure 4. Partial deletion of *TPX2-5* gene by CRISPR in the wild-type background does not lead to cell-division defects.

(a) Schematic representation of *TPX2-5* exons, position of gRNAs and gene region targeted for deletion. **(b)** Results of genotyping PCR of control (GH) with expected band size 5306 bp and two independent colonies with large gene deletion after CRISPR (expected band size ~2700 bp): *TPX2-5 Cr#30* (#30) and *TPX2-5 Cr#33* (#33). Experiment was repeated 2 times with similar results. **(c)** Representative moss colonies and individual gametophores after 4 weeks of culture. Experiment was repeated 2 times with similar results. Bars, 1 mm. **(d)** Representative fluorescence images of cell division in apical protonema cells in *TPX2-5 Cr#30* and *TPX2-5 Cr#33*. Time 0 (min) was set at NEBD. Experiment was repeated 3 times with similar results. Bar, 10 µm. **(e)** Representative fluorescence images of the first cell division in gametophore initials in *TPX2-5 Cr#30* and *TPX2-5 Cr#33*. Cell borders are outlined in yellow. Experiment was repeated 3 times with similar results. Bar, 10 µm.

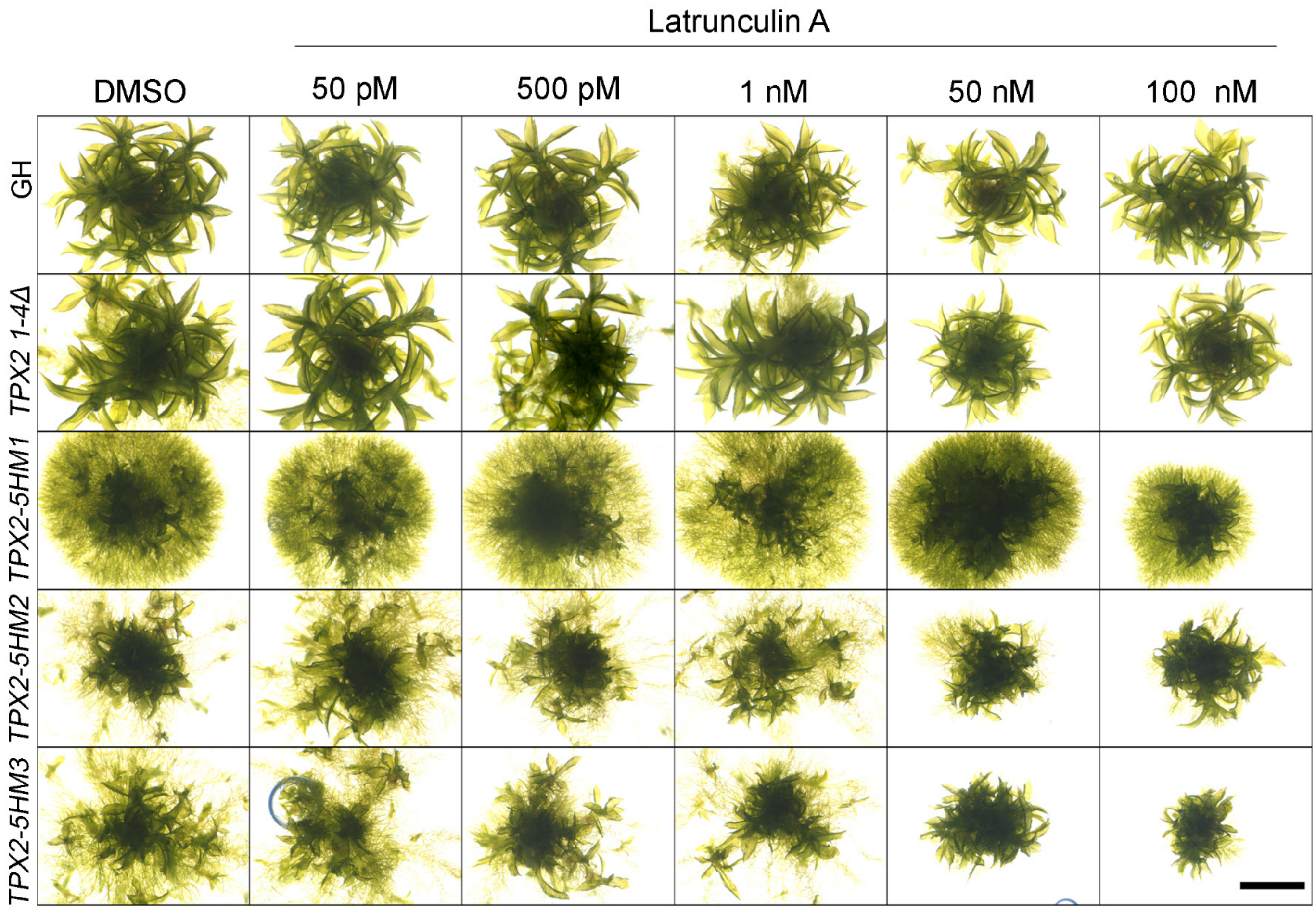

Supplementary Figure 5. Prolonged latrunculin A treatment does not mitigate stunted gametophores in *TPX2-5* hypomorphic mutants.

Representative images of moss cultured on agar plates containing DMSO or latrunculin A (50 pM to 100 nM) for 4 weeks. Experiment was repeated 2 times with similar results. Bar, 5 mm.

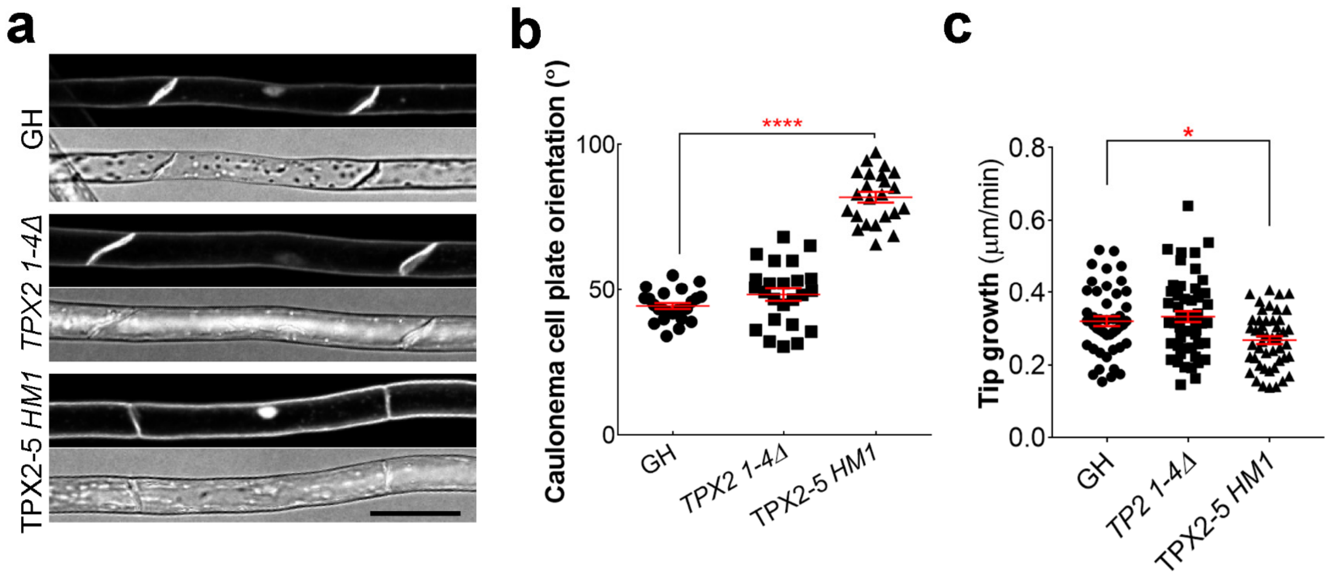

Supplementary Figure 6. Phragmoplast orientation and tip growth defects in the caulonemal tip cells in the *TPX2-5 HM1* line

(**a**) Representative images of cell plates in caulonemal cells stained with 10 µM FM4-64 dye. Experiment was repeated 2 times with similar results. Bar, 50 µm. (**b**) Acute angle of caulonemal cells in GH (control), *TPX2 1-4Δ* and *TPX2-5 HM1* lines, *n* = 24, 24, and 23, respectively (mean±SEM, **** p = 0.0001 one-way ANOVA with Dunnett's multiple comparisons test against GH). (**c**) Tip growth rate of caulonemal cells in GH, TPX2 1-4Δ and TPX2-5 *HM1* lines, *n* = 46, 50 and 48, respectively (mean±SEM, * p = 0.0144 one-way ANOVA with Dunnett's multiple comparisons test against GH).

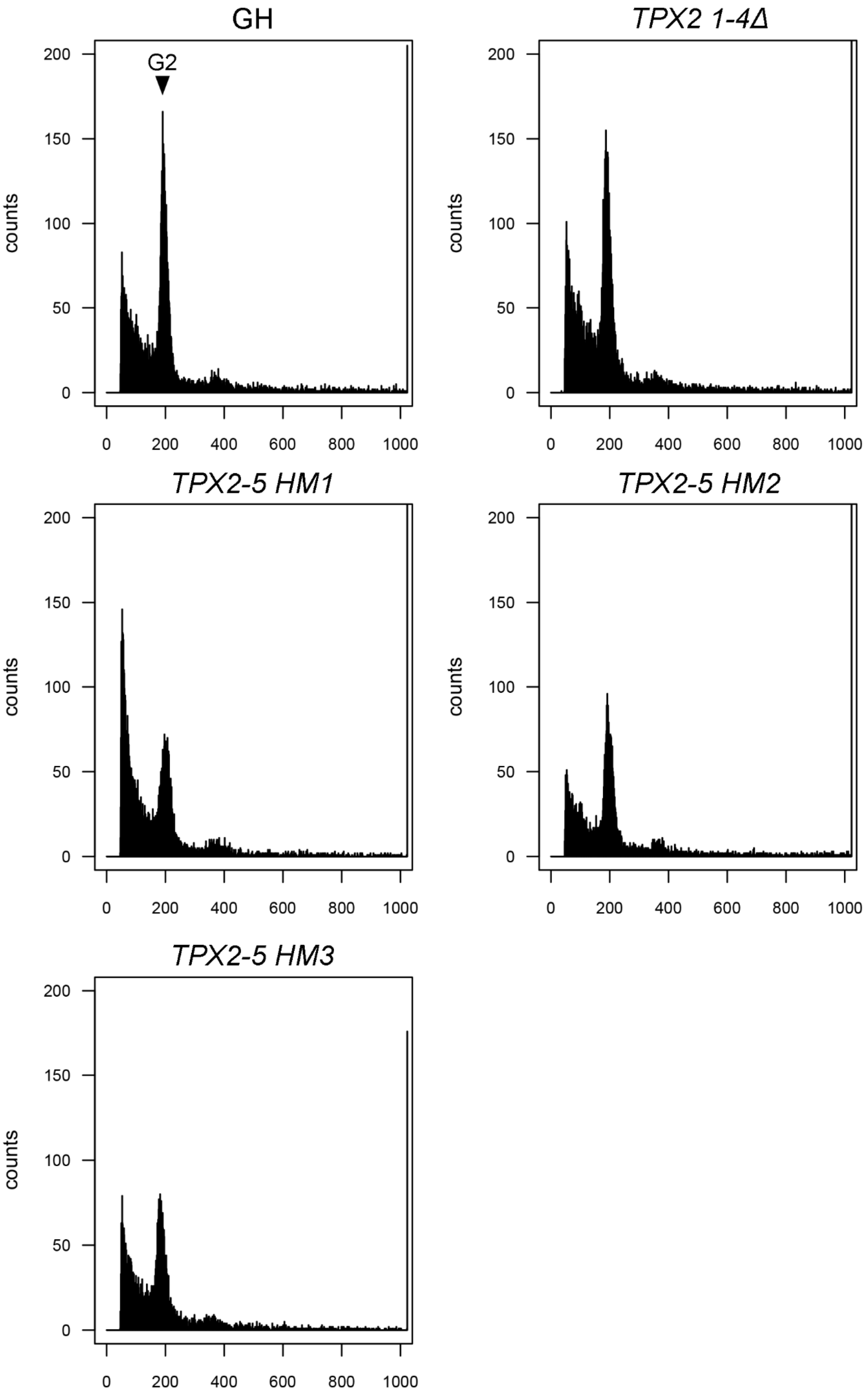

Supplementary Figure 7. Ploidy analysis of protonema tissue based on nuclei staining with DAPI

Histograms of nuclei isolated from 6-day-old protonema tissues stained with DAPI. Peak corresponding to G2 (2N) in the control line (GH) is indicated by an arrow.

Supplementary Movie 1. Localization of TPX2 proteins during mitosis
Live-cell imaging was performed in *P. patens* apical caulonemal cells expressing mCherry-tubulin (magenta) and one of the following tagged proteins (green): TPX2-1-Citrine, TPX2-2-mNeonGreen, mNeonGreen-TPX2-4, or TPX2-5-mNeonGreen. Images were acquired every 30 s in a single focal plane. Bar, 10 µm.

Supplementary Movie 2. 3D projection of gametophore initial cells after the first division. Gametophore initials were stained with 10 µM FM4-64. Cell segmentation was done with PlantSeg. The apical cell of the gametophore initial is shown in pink, while the basal cell is shown in green. Bar, 20 µm.

Supplementary Movie 3. Spindle motility underlies the erroneous phragmoplast positioning in *TPX2-5 HM1* gametophore initial cells
Live-cell imaging was performed in *P. patens* gametophore initial cells expressing mCherry-tubulin and histone H2B-mCherry (chromosomes and MTs are labeled with the same color). Images were acquired as a z-stack (20 µm in 2.5 µm steps) every 5 min, and the best focal plane was presented. Bar, 10 µm.

Supplementary Movie 4. Spindle positioning defects in later gametophore development in the *TPX2-5 HM1* mutant.
Representative video of spindle misorientation and/or motility in *TPX2-5 HM1* gametophore initial cells during the second (left cell) or later (right cell) cell divisions. The spindle axis is indicated by a cyan line. Images were acquired as a z-stack (20 µm, 2.5 µm step) every 5 min, and the best focal plane was presented. Bar, 10 µm.

Supplementary Movie 5. Actin distribution during spindle motility in the *TPX2-5 HM1* mutant.
Live-cell imaging of Citrine-F-tractin (actin, green) during the first asymmetric division in the gametophore initial also expressing mCherry-tubulin (magenta, MTs). *TPX2-5 HM1* cells additionally express mCherry-tubulin/H2B-RFP (magenta, chromosomes). Images were acquired as a z-stack (20 µm in 2.5 µm steps) every 5 min. Top panel shows merged images of maximum intensity projection of Citrine-F-tractin (green) and single best focal frame of mCherry-tubulin (magenta, wild-type) or mCherry-tubulin/H2B-RFP (magenta, *TPX2-5 HM1*). Middle panel shows merged images of best focal plane for Citrine-F-tractin (green) and mCherry-tubulin (magenta, wild-type) or mCherry-tubulin/H2B-RFP (magenta, *TPX2-5 HM1*). Bottom panel shows best focal plane for Citrine-F-tractin (gray). Bar, 10 µm

Supplementary Movie 6. Spindle-collapse phenotype in the gametophore initial of *TPX2-5 HM1* mutants
Representative video of spindle collapse followed by chromosome missegregation, observed in approximately 10% of *TPX2-5 HM1* gametophore initial cells. Images were acquired as a z-stack (20 µm, 2.5 µm step) every 5 min, and the best focal plane was presented. Bar, 10 µm.

Supplementary Movie 7. Mitotic defects in the *TPX2-5* RNAi lines
Representative images of mitotic defects in the *TPX2-5* RNAi lines. Live-cell imaging was performed in *P. patens* protonemal apical cells expressing GFP-tubulin (green) and histone H2B-mCherry (magenta). Images were acquired at a single focal plane every 2 min. Bar, 10 µm.

Supplementary Data 1. Plasmids and primers used in this study

Supplementary Data 2. Transgenic Physcomitrella lines used in this study
