# Supplementary material for: Spindle motility skews division site determination during asymmetric cell division in Physcomitrella
Source: Nat Commun. 2022 May 5;13:2488. doi: 10.1038/s41467-022-30239-1 (PMC9072379; doi:10.1038/s41467-022-30239-1)
Supplement: Supplementary file 3 — Description of Additional Supplementary Files [file 41467_2022_30239_MOESM3_ESM.pdf]

Supplementary Data 1. Plasmids and primers used in this study

Supplementary Data 2. Transgenic Physcomitrella lines used in this study

Supplementary Movie 1. Localization of TPX2 proteins during mitosis
Live-cell imaging was performed in *P. patens* apical caulonemal cells expressing mCherry-tubulin (magenta) and one of the following tagged proteins (green): TPX2-1-Citrine, TPX2-2-mNeonGreen, mNeonGreen-TPX2-4, or TPX2-5-mNeonGreen. Images were acquired every 30 s in a single focal plane. Bar, 10 µm.

Supplementary Movie 2. 3D projection of gametophore initial cells after the first division. Gametophore initials were stained with 10 µM FM4-64. Cell segmentation was done with PlantSeg. The apical cell of the gametophore initial is shown in pink, while the basal cell is shown in green. Bar, 20 µm.

Supplementary Movie 3. Spindle motility underlies the erroneous phragmoplast positioning in *TPX2-5 HM1* gametophore initial cells
Live-cell imaging was performed in *P. patens* gametophore initial cells expressing mCherry-tubulin and histone H2B-mCherry (chromosomes and MTs are labeled with the same color). Images were acquired as a z-stack (20 µm in 2.5 µm steps) every 5 min, and the best focal plane was presented. Bar, 10 µm.

Supplementary Movie 4. Spindle positioning defects in later gametophore development in the *TPX2-5 HM1* mutant.
Representative video of spindle misorientation and/or motility in *TPX2-5 HM1* gametophore initial cells during the second (left cell) or later (right cell) cell divisions. The spindle axis is indicated by a cyan line. Images were acquired as a z-stack (20 µm, 2.5 µm step) every 5 min, and the best focal plane was presented. Bar, 10 µm.

Supplementary Movie 5. Actin distribution during spindle motility in the *TPX2-5 HM1* mutant.
Live-cell imaging of Citrine-F-tractin (actin, green) during the first asymmetric division in the gametophore initial also expressing mCherry-tubulin (magenta, MTs). *TPX2-5 HM1* cells additionally express mCherry-tubulin/H2B-RFP (magenta, chromosomes). Images were acquired as a z-stack (20 µm in 2.5 µm steps) every 5 min. Top panel shows merged images of maximum intensity projection of Citrine-F-tractin (green) and single best focal frame of mCherry-tubulin (magenta, wild-type) or mCherry-tubulin/H2B-RFP (magenta, *TPX2-5 HM1*). Middle panel shows merged images of best focal plane for Citrine-F-tractin (green) and mCherry-tubulin (magenta, wild-type) or mCherry-tubulin/H2B-RFP (magenta, *TPX2-5 HM1*). Bottom panel shows best focal plane for Citrine-F-tractin (gray). Bar, 10 µm

Supplementary Movie 6. Spindle-collapse phenotype in the gametophore initial of *TPX2-5 HM1* mutants
Representative video of spindle collapse followed by chromosome missegregation, observed in approximately 10% of *TPX2-5 HM1* gametophore initial cells. Images were acquired as a z-stack (20 µm, 2.5 µm step) every 5 min, and the best focal plane was presented. Bar, 10 µm.

Supplementary Movie 7. Mitotic defects in the *TPX2-5* RNAi lines

Representative images of mitotic defects in the *TPX2-5* RNAi lines. Live-cell imaging was performed in *P. patens* protonemal apical cells expressing GFP-tubulin (green) and histone H2B-mCherry (magenta). Images were acquired at a single focal plane every 2 min. Bar, 10 μm.
